# Mesenchymal Stem Cells in Myeloid Malignancies: A Focus on Immune Escaping and Therapeutic Implications

**DOI:** 10.1155/2017/6720594

**Published:** 2017-08-21

**Authors:** Nicola Stefano Fracchiolla, Bruno Fattizzo, Agostino Cortelezzi

**Affiliations:** UO Onco-Hematology, Fondazione IRCCS Ca' Granda Ospedale Maggiore Policlinico, Via F. Sforza 35, 20100 Milano, Italy

## Abstract

The importance of the bone marrow microenvironment forming the so-called niche in physiologic hemopoiesis is largely known, and recent evidences support the presence of stromal alterations from the molecular to the cytoarchitectural level in hematologic malignancies. Various alterations in cell adhesion, metabolism, cytokine signaling, autophagy, and methylation patterns of tumor-derived mesenchymal stem cells have been demonstrated, contributing to the genesis of a leukemic permissive niche. This niche allows both the ineffective haematopoiesis typical of myelodysplastic syndromes and the differentiation arrest, proliferation advantage, and clone selection which is the hallmark of acute myeloid leukemia. Furthermore, the immune system, both adaptive and innate, encompassing mesenchymal-derived cells, has been shown to take part to the leukemic niche. Here, we critically review the state of art about mesenchymal stem cell role in myelodysplastic syndromes and acute myeloid leukemia, focusing on immune escaping mechanisms as a target for available and future anticancer therapies.

## 1. Introduction

Bone marrow stromal cells include mesenchymal stem cells (MSCs), adipocytes, osteoblasts, fibroblasts, endothelial cells, tissue macrophages, and osteoclasts. Recent evidences support the notion that patients with myeloid malignancies may present bone marrow microenvironment alterations in terms of abnormal hematopoietic-to-stromal cell interactions, relative deficiency of hematopoietic growth factors, and aberrant release of inhibitors [1]. Nevertheless, the level of MSC involvement in myeloid malignancies remains controversial. MSC molecular and genetic alterations in this context have been demonstrated, and cytogenetic abnormalities in MSC derived from myeloid malignancy patients have been reported [2–4], while other studies [5] failed to find any significant quantitative or qualitative alterations in myelodysplastic syndrome- (MDS-) derived MSCs.

Leukemogenesis is the result of multistep alterations involving both the genetic and the epigenetic levels; moreover, the immune system, far to be an innocent bystander, plays an active role in leukemic immune escaping mechanisms. In addition, it has not been completely elucidated whether cancer-associated MSCs belong primarily to the abnormal clone or emerge after leukemic stem cell induced environmental damage.

We therefore aimed to synthetically describe the state-of-the-art MSC alterations in myelodysplastic syndromes and acute myeloid leukemia, focusing on biological evidences about MSCs pathophysiologic role in immune escape, that may represent a possible target both for present and future anticancer therapies.

## 2. Mesenchymal Stem Cell Physiology

MSCs are adult multipotent cells that can be isolated from the bone marrow, umbilical cord blood, placenta, or adipose tissue [6] and represent fundamental actors in the formation, organization, and function of the hematopoietic niche [7–9]. Given their heterogeneity, the International Society for Cellular Therapy (ISCT) position statement suggested to use the term “mesenchymal SCs” only for cells that are plastic adherent in culture and express CD73, CD90, and CD105, but not CD14, CD34, CD45, CD79*α*, and human leukocyte antigen-D-related (*HLA*-*DR*). Moreover, they should be able to differentiate fibroblasts, osteoblasts, adipocytes, and chondroblasts in vitro [10] and to transdifferentiate tissues of neuroectodermal origin such as neurons or glial cells. MSC tissue of origin remains a matter of debate, and both mesodermal and neuroectodermal embryonal sheets are possible candidates [11]. The vascular tissues may be considered as a source of MSCs, as it is well known that marrow endothelial cells protect and maintain the repopulating capacities of hematopoietic precursors *in vivo* [12] and regulate proliferation and differentiation by tight spatial colocalization with perivascular cells [13] and through E-selectin secretion [14]. Cytokine and chemokine release [15] and crosstalk molecules expression, such as Jagged1 and CXCL12 [16–18], play important roles in the regulation of these interactions.

MSCs display systemic immunoregulatory and immunosuppressive properties [19–24] and influence both adaptive and innate immune responses. One of the immunomodulatory mechanisms is the expression of cell surface molecules with immunosuppressive capacity, such as programmed death ligand 1 (PD-L1) and Fas ligand, on MSC surface, so that they are able to directly deliver inhibitory signals to immune cells expressing PD-L1 and/or Fas, via cell-to-cell contact mechanisms [25, 26]. In fact, MSCs can repress Th1 and Th17 polarization [27, 28] via PD-L1 upregulation/constitutive expression [29]. In this context, their impairment has been implicated in tumor immune escaping, as described below.

Moreover, it seems that MSCs may inhibit erythropoiesis in favor of myeloid differentiation, through soluble factor production [30], including interleukin (IL) 6, which was shown to expand myeloid progenitors blocking erythroid development [31]. In this context, elevated IL-6 and TNF*α* levels have been correlated with adverse survival in patients with acute myeloid leukemia (AML) [32]. Another player engaged in the niche regulation is the autonomic nervous system that accompanies marrow blood vessels through adrenergic fibers. An interaction of adrenergic fibers with the MSC microenvironment has been described, and deregulation of this system has been implicated in impaired hematopoiesis which is a hallmark of several hematologic diseases [32, 33].

## 3. Mesenchymal Stem Cell Behaviour in Myeloid Malignancies

MSC role in MDS and AML, the two overlapping/evolving models of myeloid malignancies, will be discussed focusing on these two biological and clinical conditions which, although very similar and belonging to a unique disease spectrum, show deep differences in both cellular/molecular and outcome aspects. Recent evidences, reviewed by Pleyer et al. [34], show that MDS cells are heavily dependent on their “dysplastic niche.” MDS-derived MSCs display enhanced supportive capacities for clonal hematopoiesis by decreased expression of cell surface molecules [35], including CD44 and CD49e (*α*5-integrin) [36]. Moreover, in patients with low-risk disease displaying a hypermethylated phenotype, circulating endothelial precursors are increased and exhibit downregulation of members of the wingless-int (Wnt) signaling pathway and failure to adequately sustain normal hematopoiesis [37]. Keeping on with endothelial-associated MSCs, CD271-positive MSCs are increased in MDS/AML marrow, especially in low-risk MDS compared to high-risk MDS or AML, and show higher CXCL12 expression. CXCL12-expressing cell density, in turn, correlated with marrow blast counts and disease progression [38]. Finally, the TGF*β* pathway is constitutively activated in marrow blasts from patients with MDS, suggesting TGF*β* implication in the pathogenesis of the dysplastic niche [39]. As described for MDS, in AML, MSC-derived endothelial cells are significantly increased, especially in cases with rapidly proliferating disease, further suggesting MSCs derived cell implication in leukemic niche building. Furthermore, AML blasts have been shown to modulate endothelial cell expansion, proliferation, and activation through the upregulation of E-selectin adhesion molecule [40]. AML blasts may then adhere to the stroma and be sequestered in a quiescent status becoming chemo-resistant, constituting a pool of residual disease which will possibly lead relapse [41]. Adhesion and chemotaxis have been also evaluated in a recent study, where AML MSCs from AML patients showed similar *β*1 integrin, CD44, CD73, CD90, and E-cadherin but decreased monocyte chemoattractant protein-1 levels compared to MSCs from healthy donors. AML MSCs showed chromosomal aberrations, but no significant differences in gene expression were detected [42]. Moreover, AML MSCs display constitutive TGF*β* signaling, which may be inhibited by the transcription factor FOS upregulation, and secrete lower levels of IL-6 and granulocyte macrophage colony-stimulating factor levels, resulting in diminished supportive capacity for healthy marrow precursors [40]. Keeping on with MSC interaction with AML blasts, it has been shown that both cells constitutively release several soluble mediators, and when cocultured normal MSCs had an antiapoptotic and growth-enhancing effect on primary human AML cells in 51 unselected AML patients, this was associated with increased phosphorylation of mTOR and its downstream targets. The authors concluded that the cytokine-mediated effect of the MSCs is growth enhancement/apoptosis inhibition [43]. A recent interesting finding is that different cytogenetic/clinical AML subsets may show differences also in MSC niche, as elegantly demonstrated by Rodrigues Lopes et al., who characterized MSC cytokine expression in patients with AML with myelodysplasia-related changes (MRC), a well-recognized clinical subtype of secondary AML, and de novo AML. They found that AML-MRC MSCs presented higher IL-6 expression, whereas de novo AML MSCs presented increased expression of VEGFA, CXCL12, RPGE2, IDO, IL-1*β*, IL-6, and IL-32, and decreased IL-10 expression. Interestingly, IL-32 was shown to promote stromal proliferation, chemotaxis, and crosstalk AML blasts [44].

Finally, altered adrenergic regulation is observed in leukemic niche and this AML-induced neuropathy (i.e., sympathetic denervation of marrow arterioles and reduced sympathetic tone) reinforces leukemia progression through depletion of arteriole-associated pericytic mesenchymal cells [45].

## 4. MSC Molecular Pathway Alterations in MDS and AML

As described in murine experimental models, microenvironment molecular alterations may contribute to the induction of hematopoietic disorders. Some examples are the activating mutation in *β*-catenin and the deletion of miRNA processor gene DICER1 in osteoblasts that resulted in the development of MDS and AML in mice [46, 47]. Interestingly, as reported by Diaz de la Guardia et al., marrow MSCs, although clearly linked to disease activity and treatment outcome, do not carry tumor-specific cytogenetic/molecular alterations [48]. Recently, von der Heide et al. [2] studied genetic, transcriptional, and DNA methylation alterations in human AML MSCs and observed a nonspecific mutation pattern with variable frequency of synonymous and nonsynonymous single-nucleotide variations as well as insertions/deletions of specific genes. Interestingly, the number of variants per sample lowered from diagnosis to relapse. The only mutation with high variant allele frequency involved the PLEC gene (R1801Q), encoding the cytoskeleton linker protein plectin. PLEC mutation analysis confirmed that it is present at an early stage of AML-MSC expansion. Genome-wide DNA methylation analysis also showed globally altered DNA methylation pattern in AML MSCs. Generally, CpG methylation showed skewing toward hypomethylation in AML MSCs. Other authors [49] also reported transcriptional alterations in MDS MSCs using RNAseq analyses, contributing to multiple pathway deregulation, including adhesion molecules and metabolic pathways as well as endocytosis [50–54].

Another process involved in MSC leukemic phenotype acquisition is that of autophagy that is responsible of senescent cell molecule elimination and turn over. Reduced expression of autophagy genes was found in human AML blasts, whereas autophagy pathway is upregulated in blasts from low-risk MDS cases [55–58], suggesting a role in preventing progression to high-risk disease or AML evolution.

The gene TWIST, a transcriptional regulator contributing to MSC self-renewal and differentiation, was shown to be upregulated in MDS human blasts, and its expression was demonstrated to be altered by stroma contact and to correlate with disease stage and p53-mediated apoptosis [59–61]. In this context, TWIST tumor suppressor function, exerted by p21 activation, appears epigenetically silenced by hypermethylation in 31% of adult AML patients, providing leukemic cells with proliferation and survival advantages [62].

Finally, Notch/Jagged1 abnormalities have been described in human MSCs, leading to impaired differentiation and plasticity and contributing to MDS pathogenesis [63], while constitutively active *β-catenin* expression favored AML induction in murine models [46]. Moreover, Geyh et al. [64] showed that MSCs from AML patients exhibit Kit-ligand and Jagged1 pathway alterations, causing growth deficiency and impaired osteogenic differentiation capacity, partially reversible and correlating with a disease status.

## 5. MSCs and Tumor Immune Escape

### 5.1. Immune System in Myeloid Malignancies: A Naive Surveillant?

Innate and adaptive immune pathways demonstrate aberrant activation in the hematopoietic niche of MDS playing a role in the increased rate of apoptosis of marrow precursors which is the hallmark of marrow failure. Moreover, an increased incidence of autoimmune diseases (e.g., primary immune thrombocytopenia, vasculitis, hypothyroidism, and rheumatoid arthritis) in patients with MDS has been reported in epidemiological studies [65], and recently, [66] the existence of a spectrum of pathophysiologic entities, spanning from chronic autoimmune attack to frank MDS and AML, through idiopathic cytopenia/dysplasia of undeterminate significance (ICUS/IDUS), has been hypothesized. Focusing on cellular immunity, naïve T cells (CD3+) exhibit shorter telomere length and have significantly less proliferative potential in MDS than in normal controls; oligoclonal T cells, often derived from the malignant MDS clone, may be present and seem to inhibit hematopoiesis *in vitro,* possibly through MHC class I molecules targeting on benign and malignant hematopoietic precursors, as a part of tumor surveillance. As the targeting of these mechanisms through immune-suppressive therapies has not been associated to AML progression, it is thought that MDS cells escape immune patrolling through a cytokinic permissive stromal pattern. These aspects are also shared byaplastic anemia, another genotypical (as these patients harbor mutation characteristic of MDS and ICUS/IDUS in almost half of cases) and phenotypical models bridging autoimmunity and malignancy, in which T cell-mediated marrow suppression leads to bone marrow failure with an increased risk of AML evolution [67]. Altogether, these alterations may reduce the “naive” immune surveillance on malignant transformation, modulating those mechanisms that, although various and articulated, may be eluded. In MDS patients, an increased expression of inflammatory T helper 1 cytokines has been reported, contributing to systemic symptoms and increased apoptosis [67]. In particular, tumor necrosis factor alpha (TNF-*α*) overexpression is inversely correlated to hemoglobin and survival, as it favors Fas-mediated- and TNF-related apoptosis-inducing ligand- (TRAIL-) driven ineffective hemopoiesis. Moreover, IFN regulatory factor 1 (IRF-1) mRNA, a tumor suppressor gene involved in T cell maturation and inflammatory responses, has been found upregulated in MDS patients with autoimmune phenomena compared to those without [68, 69]. Transforming growth factor-*β* (TGF-*β*) has been implicated in MDS hemopoietic suppression directly or mediated by the production of other myelosuppressive cytokines (e.g., IL-6, IL-32, IFN-g, and TNF), leading to decreased B cell proliferation, natural killer dysfunction, and propagation of cell autophagy or apoptosis. Inhibition of TGF-*β* receptor I kinase was shown to decrease apoptosis and improve erythroid and myeloid colony formation in vitro. T-helper 17, involved in the development/prevention of autoimmunity and inflammation, is significantly increased in low-risk MDS [69]. Finally, innate immunity is highly active in MDS cases due to overexpression of TLR activators, such as MYD88, TIRAP, IRAK1/4, and TRAF, and downregulation of inhibitory factors, such as microRNAs miR145 and miR146a [70].

In AML, the immune system may play a role in enabling blast proliferation originating from the leukemic stem cell which in turn escapes surveillance. Cellular-mediated immune killing is driven by T CD8+ recognition of aberrant/exogenous molecules presented on MHC Class I complex, and the loss/mutation of MHC Class I is a demonstrated escape strategy in solid tumours. In AML, MHC Class I loss is rare, but allows NK cells to escape through killer cell immunoglobulin-like receptors (KIR). Moreover, expression of nonclassical HLA molecules, as reported for soluble HLA-G detected in AML sera, may contribute to immune suppression. CD4+ T cells and antibody-driven immunity rely on MHC Class II recognition of presented extracellular antigens; these molecules present variable expression on AML cells, and their total loss on promyelocytic leukemia cells may represent a possible escaping strategy. Currently, great interest is being given to AML-associated antigens (e.g., antigens aberrantly expressed by AML cells) that may elicit immune responses. For example, Wilms' tumour protein 1 (WT1), a zinc finger transcription factor overexpressed in AML cases, especially in leukemic stem cells, provides a target to eliminate the quiescent neoplastic cells. Moreover, some AML-specific genetic alterations may lead to aberrant antigen recognition through both MHC Classes I and II, as observed for FLT3-ITD, PML-RARalpha, BCR-ABL, DEK-CAN, and NPM1 mutations. As described for MDS, both innate and adaptive immunities are eluded by AML blasts through different mechanisms: (1) suppression of NK cell-mediated cytotoxicity by the inability to lyse AML cells, production of cytotoxic cytokines, aberrant expression of VEGFC, and by immature NK cell inhibition of T cell activation. (2) T cell suppression through aberrant gene expression in T cells, through the inability to form effective immune synapses with AML blasts, and by the coexpression of immunosuppressive proteins (TIM-3 and PD-13). Higher proliferation of Tregs facilitates blast expansion by T effector cell suppression through secretion of immunosuppressive cytokines (e.g., TGF-beta). In addition, Tregs increase the production of adenosine, an immunosuppressant for T and NK cells. (3) Secretion/expression of immunosuppressive factors by AML cells, including indoleamine 2,3-dioxygenase 1 (IDO) expression, arginine metabolism, and secretion of reactive oxygen species [71]. It is noteworthy to mention that immunologic milieu in AML is dynamic, as it is altered and modulated not only by the disease itself, but even by therapeutic interventions. As a matter of fact, AML in remission after chemotherapy shows decreased innate (neutrophils and monocytes) and specific immune activities (B cell activation) and cytokine pattern may also be changed. Even deeper is the change after allogeneic transplant, where immune reconstitution is a long and complicated process implying the adoption/maturation of the donor immunocompetent cells and the persistency of the recipient memory cells. In a recent study, evaluating the functional capacity of the immune system in 10 adult patients with AML using the response to seasonal influenza vaccination as a surrogate, only 2 patients generated protective titers in response to vaccination and a majority of patients had abnormal frequencies of transitional and memory B cells, with B cell repertoire showing little evidence of somatic hypermutation. Conversely, T cell populations were similar to healthy controls, and cytotoxic T cells demonstrated antigen-specific activity after vaccination, with T-effector cells showing increased PD-1 expression with possible therapeutic implications (see specific paragraph) [72].

### 5.2. MSCs Interact with Microenvironment Immunologic Landscape

MSCs have immunoregulatory activities exerted by interaction with a large number of effector cells, including T cell subsets, B cells, NK cells, monocyte-derived dendritic cells, and neutrophils, by direct cell-to-cell adhesion and/or secretion of soluble molecules, the so-called MSC secretome, which includes soluble molecules and extracellular vesicles (EVs) released by the MSCs into the extracellular milieu. Moreover, terminally differentiated mesenchymal cells prevent both proliferation and apoptosis of activated T cells. MSC-mediated immune regulation encompasses several mediators including interferon-*γ* (IFN-*γ*), interleukin-1*β* (IL-1*β*), transforming growth factor-*β*1, indoleamine-2,3-dioxygenase (IDO), IL-6, IL-10, prostaglandin-E2, hepatocyte growth factor, tumor necrosis factor (TNF)-*α*, nitric oxide (NO), heme oxygenase-1, and HLA-G5. These suggest a wide variety of targets for both immune inhibition and escaping mechanisms. Concerning innate immunity, MSCs are able to inhibit neutrophil proinflammatory activities suppressing the respiratory burst and prolonging survival through IL-6 and STAT-3 pathways. Moreover, they inhibit mature DC differentiation while promoting IL-10-producing plasmacytoid DCs. In a recent report, bone marrow MSCs were able to inhibit mouse DC activity by decreasing expressions of TLR3 and TLR9, confirming the immunomodulatory role of MSCs in a cell-based therapy [73]. Finally, MSCs suppress NK proliferation, soluble factor production, and cytotoxic activity. As long as adaptive immunity is concerned, MSCs promote quiescent T cell survival, but induce anergy of activated T cells and inhibit proliferation in favor of Tregs development driven by IL-10 secretion. B cell proliferation/differentiation is also suppressed both directly and through activated CD4+ T cell suppression. These immunoregulatory properties are exerted both directly and through MSC-derived exosomes, which have been used for therapeutic purposes as later discussed [74]. The abilities of MSCs to modulate immune responses may contribute to their therapeutic activity in models of autoimmune/inflammatory disorders. As an example, in rheumatoid arthritis, human adipose tissue-derived MSCs were able to inhibit IL-17 and IL-21 secretions by mononuclear cells and to increase TGF-*β* expression with consistent immunomodulatory effects [75].

### 5.3. MSCs Contribute to Leukemia Permissive Niche Also by Immune-Mediated Mechanisms in Lymphoid and Myeloid Models

Tumor microenvironment is thought to be inflammatory and “educated” by the neoplastic cells to be permissive in favor of the neoplastic clone growth. MSCs from the neoplastic niche are then in turn able to switch their phenotypes from MSC Type 1 (proinflammatory) to “tumor-educated” MSC Type 2 cells (anti-inflammatory) that exhibit stronger immunosuppressive and migratory properties and drug resistance and promote proliferation [76, 77]. Interestingly, transcriptome profiling assays revealed a proinflammatory signature in bone marrow MSCs, with deregulation of immune and inflammatory modulators of the prostaglandin synthesis, largely depending on the genomic instability characteristic of oncogenic transformation, which is more typical of solid tumor than of AML, that is known to show limited number and type of identifiable driver/passenger mutations [78, 79]. The phenomenon of epithelial to mesenchymal transition, typical of solid tumors and linked to metastatization ability, is another evidence of mesenchymal contribution to tumor escape: malignant epithelial cells modify their transcriptional programme losing cell–cell attachments and acquiring mesenchymal-like features and motility, through deregulation of molecular pathways that include TGF*β*, Wnt, Notch, hedgehog, and tyrosine kinase receptors. This phenotype has been associated in solid tumors with poor prognosis and resistance to radio- and chemotherapy. A minority of these mesenchymal-switched-circulating tumor cells migrate and survive in the bloodstream, evading immune surveillance, extravasate, and are able to colonize target organs or tissues [79]. Similarly, chronic lymphocytic leukemia-derived exosomes actively promote disease progression by modulating several functions of surrounding stromal cells that acquire features of cancer-associated fibroblasts. More in details, CLL-derived exosomes are actively incorporated by endothelial and mesenchymal stem cells that show enhanced proliferation, migration, and secretion of inflammatory cytokines, contributing to a tumor-supportive microenvironment [80, 81]. Cancer-associated MSCs have greater chemotactic activity on mononuclear cells and Tregs and inhibit T cell cytotoxicity and B cells and NK cells, as observed in solid and hematologic malignancies [82–85]. As mentioned above, MSCs secrete IDO and PGE2. The IDO enzyme activates dendritic cells and macrophages, helping to create an environment that favors suppression and tolerance. This molecule has been shown to play a relevant role in promoting solid/liquid tumor tolerance in vitro and in vivo murine models [86–88]. In addition, many human tumors have been shown to produce IDO [89]. In a recent report, MSCs isolated from 20 bone marrow AML samples showed higher expression of IDO and increased Tregs compared to control subjects, with a positive correlation between IDO expression and Tregs, responsible for immunosuppressive microenvironment [90]. Neoplastic MSCs in MDS and AML decrease tumor immunosurveillance through downregulation of costimulatory molecules (CD40, CD80, and CD86) [91], increase immunosuppressive cytokine production (TGF*β*, IL-6, and HGF) with consequent T cell and DC suppression and Tregs and MSC type 2 increases. These alterations are more markedly observed in high-risk than in low-risk MDS [92, 93]. Constitutive overexpression of IDO observed in AML blast cells and sera [94] correlates with decreased relapse-free and overall survival [95]. Moreover, high IDO levels in MDS cases correlate with cytopenias severity [96].

Recent evidences support the role of T helper (Th) cells in the pathogenesis of hematological malignancies, including Th1/Th2 unbalance, increased Treg and Th17 levels, as well as the recently identified Th22 cells, that produce IL-22, which belongs to IL-10 cytokine family involved in the pathogenesis of autoimmune diseases and MDS. Tian et al., studied T helper subsets in AML patients and found that, while Th1, Th2, Th17, and Th22 cells and IL-22 were significantly reduced, Treg cells were increased in newly diagnosed AML patients compared to patients in remission or controls and chemotherapy ameliorated these variations [97]. MSC-mediated alteration of immune environment may be reversible with leukemia treatment, as also shown in patients with juvenile myelomonocytic leukemia, whose MSCs showed differential mRNA expression, including genes involved in immunomodulation and cell–cell interaction, that normalized during remission after successful hematopoietic stem cell transplantation [98].

As far as innate immunity is concerned, coculture of AML cells with MSC significantly protects leukemic blasts from NK cell-mediated lysis, mainly through cell-to-cell contact with supportive MSC, implying a relevant role of MSC in the immune response against AML blasts [99]. Moreover, overexpression of toll-like receptor- (TLR-) regulated genes has been described in MDS, resulting in excessive apoptosis with consequent cytopenias and decreased erythropoiesis in lower risk stages. In fact, TLR1, 2, and 6 expressions are higher in CD34+ blasts from patients with lower risk compared to higher risk MDS, whereas TLR2 and 4 expressions were similar in AML patients and healthy controls [100–102]. Concerning clinical correlations, higher expression of TLR2 is associated with prolonged survival, whereas higher expression of TLR6, TLR7, and MYD88 (a key mediator of TLR signaling) might confer a worse prognosis. On the contrary, in AML, stimulation of TLR2 and 4 resulted in induction of immune escape mechanisms such as upregulation of PD-L1, which protected AML cells from cytotoxic T lymphocyte lysis in vitro [103, 104]. Finally, recent evidences also show antileukemic effects for TLR8 activation, independently from its immunomodulating properties [105].

On the contrary, in lymphoid malignancies, a host beneficial immunomodulatory effect mediated by MSCs has been shown in low/intermediate risk acute lymphoblastic leukemia patients, where MSCs promoted an efficient NK cell response including cytokine production, phenotypic activation, and cytotoxicity [106].

Recently, Giallongo et al. identified a population of myeloid-derived suppressor cells (MDSC) in chronic myeloid leukemia (CML) patients that is part of the tumor clone and provides a leukemic friendly microenvironment mediated by ARG1, NOS2, reactive species of oxygen (ROS), cyclooxygenase 2 (COX2), TGF*β* and immunosuppressive cytokine production, inhibition of NK function, and Treg expansion. When MSC-educated MDSC from healthy donors and CML patients were generated by coculturing MSC with peripheral blood-mononucleated cells, only CML-MSC-educated MDSC exhibited a suppressive ability on autologous T lymphocytes and overexpressed TGF*β*, IL-6, and IL-10, thus contributing to CML immune escape [107].

## 6. Mesenchymal Stem Cells: Therapeutic Implications

### 6.1. Targeting Immune Escape

The immune disregulation of the dysplastic and leukemic niches is an interesting target for biological therapies. Recently, growing evidences support the use of immune checkpoint blockers as well as engineered immunocompetent cells and monoclonal antibody therapies engaging specific T cells in hematologic malignancies. Immune checkpoints are regulatory pathways that are induced in activated T cells and regulate the amplitude as well as the quality of T cell antigen responses. However, cancer can exploit these immune cell-intrinsic checkpoints for escaping immune-mediated destruction, by upregulation and activity of checkpoint molecules on T cells, leading to reduction or elimination of antitumor immune activity. CTLA-4 and PD-1 are two of the most actively studied inhibitory receptors expressed by activated T cells. The PD-1 pathway not only suppresses functions of effector T cells, lytic capacity of NK cells and B cell antibody production, but also promotes Treg stability and functions, thus contributing to the maintenance of immune suppression in the microenvironment. PD1/PDL1 upregulation may be targeted with immune checkpoint blockers. As a matter of fact, immune checkpoint inhibitors may enhance cytotoxicity of cytokine-induced killer cells against human myeloid leukaemic blasts [108], suggesting that also AML may benefit from checkpoint inhibition, as it has been demonstrated in solid cancers.

In a recent study, vaccination with MSCs exposed to microgravity inhibited proliferation and promoted apoptosis of tumor tissue, by inducing Th1-mediated cytokine response and CD8-dependent cytotoxic response and by increasing MHC1 and HSP protein expressions. The enhanced antitumor immune response of MSCs was strongly associated with the higher expression of MHC class I molecule on DCs that made tumor molecules more cross-presentable to the host DCs to generate protective antitumor activity [109].

Another interesting approach consisted in the adoptive transfer of gene-modified MSCs, which produce and secrete tumor-directed monoclonal antibodies continuously in the body of the patient, as bispecific autoantibodies have short half-lives *in vivo* and are rapidly cleared from circulation. As MSCs have limited immunogenicity and tend to accumulate in close proximity of the tumor, they can be used as a platform for the targeted delivery of anticancer agents. Aliperta et al. recently demonstrated that gene-modified MSCs are able to express the CD33-CD3 bispecific antibody at high levels and to mediate an efficient lysis of AML blasts by human T cells of both healthy donors and AML patients [110]. The same mechanism was explored in a murine model of disseminated ALL, to selectively deliver measle virus to leukemic cells [111]. As regards leukemic MSC role in adapted immunity, they seem to favor chemotaxis of mononuclear cells and Tregs and to inhibit T cell cytotoxicity and B cell and NK cell activities, possibly through the secretion of IDO and PGE2 [86]. This observation may provide a further rationale for the use of IDO inhibitors in myeloid malignancies. Recently, Ninomiya et al. showed that tumor microenvironment suppresses chimeric antigen receptor T (CART) cell activity through IDO immune escape in a xenograft lymphoma model. Moreover, they report that fludarabine and cyclophosphamide downregulate IDO expression and improve CART antitumor activity [112].

MSCs of MDS overexpress TLR-regulated genes, resulting in excessive apoptosis with consequent cytopenias and decreased erythropoiesis. Preliminary *in vitro* observations favour the rationale of targeting aberrant TLR signaling in this setting [113]. Furthermore, TLR2 and 4 are upregulated in AML, resulting in induction of immune escape mechanisms such as upregulation of PD-L1, which protects AML cells from cytotoxic T lymphocyte [114], with a negative prognostic impact. Consequently, TLR agonists have been used as immunotherapy with the intent of inducing blast-derived dendritic cell maturation, both in vitro [115–119] and in clinical trials [120].

As regards antibodies and antibody-derived agents such as antibody drug conjugates and bispecific agents, CD33 is a valuable and clinically validated target; a prototypical agent, gemtuzumab ozogamycin, was shown to be effective in AML treatment, but was withdrawn from the clinical use because of side toxicities, mostly due to the chemical linker connecting the toxin component to the antibody carrier, even if in early 2017 a new request for US and EU approval was submitted. Moreover, antibodies specific for CD123 are under evaluation and seem to target the leukemia stem cells. New CD33-directed agents include new toxins coupled with improved linkers to the antibody (e.g., SGN-CD33a), bispecific antibodies recruiting effector cells like NKs or T cells (e.g., AMG-330), and single-chain triplebodies (e.g., 123–16-33 and 33–16-33) with a double receptor for the AML blast and one recruiting NKs. Immune reconstitution has a key role in enabling immune-mediated therapies in AML patients in remission to eliminate leukemic stem cells and MRD. NKs recovered from AML patients at diagnosis are often reduced and show reduced cytolytic activity; a recent study compared blood titers and cytolytic function of NKs from an AML patient with those of a healthy monozygotic twin. The Authors found that NK functions measured in the AML patient after disease remission were comparable to those of his healthy twin brother; moreover, ex vivo cytolytic activities mediated by triplebody SPM-2 were also maintained, making triplebodies promising new agents for the treatment of AML [121].

Graft versus host disease (GVHD) has been one of the autoimmune targets for MSC therapies in mouse models. These models are unfortunately different from the human counterparts as murine MSCs are more prone to spontaneous immortalization and transformation and IDO is not involved in mouse MSC-mediated immunoregulation. In mice, a single infusion of MSCs at the time of transplant does not prevent GVHD, whereas multiple injections may be beneficial, and engraftment of MSCs at GVHD sites is low. MSC cotransplantation does not promote engraftment in T cell-replete transplants, but might be beneficial in T cell-depleted transplants. MSC treatment of corticosteroid-refractory acute GVHD showed controversial results in human, with a European trial providing 30 out of 55 complete responses, whereas another study failed to demonstrate any durable benefit. Recently, a phase II randomized study showed that repeated infusion of MSCs may inhibit chronic GVHD symptoms and reverse the Th1/Th2 cell ratio imbalance [74].

An interesting therapeutic approach is adoptive immunotherapies in myeloid malignancies that include, among others, bone marrow allogeneic transplant, donor lymphocyte infusion (DLI), and chimeric antigen receptor T (CART) cells [122]. These approaches are currently aimed at leukemic blast targeting, but MSC patterns might be new targets in the next future. In particular, CART cells, engineered with synthetic polypeptides consisting of an extracellular variable fragment directed to a tumor antigen and an intracellular signaling domain, potentiated by the addition of costimulatory molecules in new generation models, have shown great success in relapsed/refractory ALL and may find a role in AML too, possibly targeting AML-associated/specific antigens like CD33, CD123, Lewis Y antigen, and folate receptor beta [123]. DLI is effective in reinducing response in AML residual/relapsing disease after allogeneic stem cell transplant and is currently investigated as a way to eradicate minimal residual disease (MRD), which correlates to higher relapse rate and reduced survival in AML [124].

Considering MDS, the therapeutic efficacy of lenalidomide is of great interest and is being moved from 5q syndrome to all low-risk cases. The drug, while directly inducing proliferation inhibition and apoptosis of MDS cells, plays important immunomodulatory activities by inducing T cell-mediated cytotoxicity, through CD28 costimulation and interferon-*γ*- and interleukin-2-increased productions, and by NK cell activation. At the same time, lenalidomide decreases inflammation by inhibiting the production of proinflammatory cytokines such as TNF-alfa and interleukin-1,-6,-12 and eliciting anti-inflammatory cytokine release, like interleukin-10, thus contrasting the inflammatory microenvironment that is detrimental to normal hematopoiesis [125].

### 6.2. Other MSC-Related Targets

The observation that cell-to-cell contact is necessary for “stemness” maintenance has led to the evaluation of adhesion mechanisms as possibly targetable players in malignant bone marrow milieu. In this context, signaling pathway of adhesion protein HSP90*α*/*β* has been shown to give proliferative advantage to MDS MSCs in patients with advanced-stage MDS, [126] being a potential therapeutic target in MDS. A recent study demonstrated the inhibitory effects of BIIB021, an orally available Hsp90 inhibitor, on an imatinib-resistant chronic myeloid leukemia cell lines, with significant growth inhibition and apoptosis and autophagic response [127]. HSP90 also safeguards proteins and is deacetylated by histone deacetylases 6; recent data show that histone deacetylase inhibitor-induced acetylation of HSP90 might control oncologically relevant proteins, especially in leukemic cells [128]. Moreover, various experiments indicate that [129, 130] CD44 adhesion pathway is of great interest in human AML and therapeutic blocking of this molecule in AML cells has been evaluated in murine xenografts, with some promising results [131]. For example, Li et al. reported that the anti-CD44 monoclonal antibody A3D8 inhibited proliferation of acute leukemia cell line HL-60. The A3D8 treatment increased the percentage of G0/G1 cells [132]. However, other in vitro experiments showed that MSCs may escape this targeted therapy and that leukemic stem cells become less microenvironment dependent in advanced-stage AML, so that targeting of CD44 may be less successful than expected. Anyway, some phase 1 trials are testing antibody against CD44v6 (bivatuzumab mertansine) in solid tumors, and MDS/AML setting might be a future field of application [133, 134].

As described in a recent study from von der Heide et al. [2], plectin, a cytoskeleton linker protein, is highly overexpressed in AML BM MSCs and its mutations are present at diagnosis, at the time of response and at relapse. This protein is also a biomarker for pancreatic ductal adenocarcinoma cells and a recent study [135] designed a plectin-1 selective drug delivery to pancreatic malignant cells. As immunohistochemical staining of plectin in bone marrow showed high proportion of plectin-positive cells in AML, a rationale may exist for such therapeutic strategies in this disease too. The targeted drug delivery that uses MSC for the spatial control of drug release is a growing field that will possibly have further implication in AML niche-directed therapies.

One of chemotaxis is another potentially targetable pathway: inhibition of CXCL12 stromal cells, and/or the CXCR4/CXCL12 axis, has shown promising results in mice and is currently being explored in clinical trials of MDS and AML [136–139]. Peng et al. report that a humanized anti-CXCR4 monoclonal antibody, LY2624587, blocked SDF-1 binding to CXCR4 in human lymphoma and leukemia cells, inhibiting cell migration, cell signaling including activation of MAPK and AKT, and mediating receptor internalization and CXCR4 downregulation on the cell surface. LY2624587 caused dose-dependent apoptosis in vitro and in mouse xenograft models, providing significant survival benefit [140]. Besides receptor antagonists that directly inhibit leukemic cell proliferation, preclinical and clinical studies demonstrate that CXCR4 inhibition mobilizes leukemic-lymphoma cells from their niches, improving conventional chemotherapy efficacy. An interesting study recently highlighted that runt-related transcription factor 3 (RUNX3) is involved in MSC-mediated protection of leukemia cells from As2O3- (arsenic trioxide-) induced apoptosis. In particular, in the presence of MSCs, As2O3-induced expression of RUNX3, modulated by CXCL12/CXCR4 signaling, was reduced. Furthermore, overexpression of RUNX3 restored the sensitivity of leukemic cells to As2O3. Therefore, RUNX3 is a promising target for therapeutic approaches to overcome MSC-mediated drug resistance [141].

Again, in the field of soluble niche molecules, high levels of TGF*β* are secreted by leukemic MSCs, possibly contributing to impaired normal hematopoiesis in MDS. The TGF*β* pathway, which is constitutively active in MDS blasts, may be targeted by specific inhibitors. Suppression of TGF*β* signaling in murine models and in human bone marrow samples in vitro was shown to be able to restore normal myelopoiesis and erythropoiesis [30, 40]. Moreover, Naka et al. demonstrated the therapeutic efficacy of EW-7197, an orally bioavailable TGF*β* signaling inhibitor, combined with tyrosine kynase inhibitors, in eliminating chronic myeloid leukemia initiating cells in vivo: the combination significantly delayed disease relapse and prolonged survival [142]. In another study, TGF-*β*-transduced MSCs were able to suppress T cell proliferation and IFN-*γ* release and to reduce expressions of CD40, CD86, and MHC II as well as TNF-*α* secretion by dendritic cells, while IL-4 secretion was enhanced. TGF-*β*-transduced MSCs could provide a promising tool for treatment of clinical conditions such as organ transplantation, GVHD, and autoimmune disorders [143].

As regards DNA methylation in myeloid malignancies, altered epigenetic modifications in myeloid blasts are thought to be highly pathogenetic in these diseases, providing a rationale for hypomethylating drug use and efficacy. These drugs form nowadays the backbone for anticancer therapies in the elderly and in those patients ineligible for intensive chemotherapy. As a matter of fact, recent studies demonstrated that leukemic MSCs harbor highly specific differences in methylation patterns in comparison to healthy volunteers, indicating differential activity and function of MSCs in these diseases. In this context, the epigenetically regulated Wnt/*β*-catenin pathway was shown to be altered in MSCs from myeloid malignancies and associated with adverse prognosis [144–146]. Hypomethylating agents are able to demethylate Wnt antagonist gene promoters in vitro [147], leading to Wnt reexpression and normal myelopoiesis restoration. Similar results were described for the above mentioned gene TWIST, implicated in the pathophysiology of clonal myeloid diseases [148], whose reexpression in vitro occurred after treatment with hypomethylating agents [149]. Regarding autophagy, which confers a sort of cell protection in lower risk MDS and becomes defective along with disease progression, it could be another possible target for hypomethylating agents. In fact, downregulation of autophagy-associated genes was shown to be due to promoter hypermethylation in higher risk MDS and AML, and reexpression of autophagy-associated genes has been observed in AML cell lines [55, 56].

## 7. Conclusions

Current knowledge highlights the importance of a leukemic permissive bone marrow niche in supporting abnormal myeloid precursor clonal proliferation. MSC- and MSC-derived cells show morphologic, cytogenetic, and molecular abnormalities in myeloid malignancies encompassing cell adhesion, cytokine signaling, autophagy, and methylation patterns, leading to and favoring differentiation arrest, proliferation, and clonal selection. Moreover, the immune system, both adaptative and innate, participates in the leukemic permissive milieu and immune escape correlates with disease relapse and refractoriness to standard therapies. Finally, these patterns are currently being investigated as suitable targets for already available and future anticancer therapies. The development of immune escape-directed treatments will have to consider the cross-talk between immune cells and MSCs, particularly considering disease characteristics, phase (onset, remission, and MRD status), immune reconstitution, and therapeutic goal.
